# Development and validation of a prenatal predictive nomogram for the risk of NICU admission in infants born to Chinese mothers over 35 years of age: a retrospective cohort study

**DOI:** 10.1186/s12884-024-06582-0

**Published:** 2024-05-27

**Authors:** Yihong Wei, Shuai Xu, Wenjuan Sun, Fanzhen Hong

**Affiliations:** Department of Obstetrical, The Second Hospital, Cheeloo College of Medicine, Shandong University, Jinan, 250012 People’s Republic of China

**Keywords:** Advanced maternal age, Neonatal intensive care unit, Pre-delivery factors, Prediction nomogram, Retrospective cohort study

## Abstract

**Background:**

The rising number of women giving birth at advanced maternal age has posed significant challenges in obstetric care in recent years, resulting in increased incidence of neonatal transfer to the Neonatal Intensive Care Unit (NICU). Therefore, identifying fetuses requiring NICU transfer before delivery is essential for guiding targeted preventive measures.

**Objective:**

This study aims to construct and validate a nomogram for predicting the prenatal risk of NICU admission in neonates born to mothers over 35 years of age.

**Study design:**

Clinical data of 4218 mothers aged ≥ 35 years who gave birth at the Department of Obstetrics of the Second Hospital of Shandong University between January 1, 2017 and December 31, 2021 were reviewed. Independent predictors were identified by multivariable logistic regression, and a predictive nomogram was subsequently constructed for the risk of neonatal NICU admission.

**Results:**

Multivariate logistic regression demonstrated that the method of prenatal screening, number of implanted embryos, preterm premature rupture of the membranes, preeclampsia, HELLP syndrome, fetal distress, premature birth, and cause of preterm birth are independent predictors of neonatal NICU admission. Analysis of the nomogram decision curve based on these 8 independent predictors showed that the prediction model has good net benefit and clinical utility.

**Conclusion:**

The nomogram demonstrates favorable performance in predicting the risk of neonatal NICU transfer after delivery by mothers older than 35 years. The model serves as an accurate and effective tool for clinicians to predict NICU admission in a timely manner.

**Supplementary Information:**

The online version contains supplementary material available at 10.1186/s12884-024-06582-0.

## Introduction

Pregnancy at advanced maternal age (AMA), which is defined as 35 years or older, has become increasingly common in China over the past 3 decades [1]. . It was previously shown that mothers of AMA are at a higher risk of having their newborns transferred to the neonatal intensive care unit (NICU) [2]. The NICU is a medical facility primarily dedicated to the treatment of critically ill newborns within the first 28 days of life. This specialized unit plays a crucial role in the effective management of neonatal asphyxia and various associated conditions, including hypoxic ischemic encephalopathy, aspiration pneumonia, meconium aspiration syndrome, and other forms of organ injury. Consequently, the presence of NICUs significantly enhances the survival prospects of newborns affected by high-risk conditions.

However, the admission of a newborn to the NICU inflicts significant psychological distress upon the mother [3] and the newborn. Research has demonstrated that children aged 4 to 11 years who have been in NICU care are nearly twice as likely to develop one or multiple mental disorders. Furthermore, these children are more susceptible to conditions such as separation anxiety disorder, specific phobias, attention deficit hyperactivity disorder, and oppositional defiant disorder [4]. The NICU is a relatively secluded ward that restricts parental accompaniment to minimize the risk of infection. Consequently, this process exacerbates the emotional strain experienced by the family. Furthermore, the quality and grade of NICU have been shown to be positively correlated with cost, thereby imposing a financial burden on families.

In recent years, China has relaxed its policies on family size, allowing families to have more children. Consequently, this has led to a significant rise in births among mothers of AMA. This demographic shift has led to increased incidences of various complications and complexities [5]. However, reliable and consistent predictive tools for assessing the likelihood of NICU admission for newborns born to older mothers are currently lacking.

The present study aims to construct a prenatal prediction model for evaluating the risk of neonatal NICU admission for infants of older mothers in order to provide valuable insight into the development and implementation of targeted interventions for mitigating the risk of such admissions.

## Materials and methods

### Study design and population

This is a retrospective study of neonates admitted to the NICU of the Second Hospital of Shandong University. The research protocol was approved by the hospital Ethics Committee, which waived the requirement for informed consent (Supplementary material 1). A total of 4252 neonates born to mothers over 35 years of age at the Second Hospital of Shandong University between January 1, 2017 and December 31, 2021 were included in the study. These newborns were divided at a 7:3 random sampling ratio into the training cohort (2953 neonates) and validation cohort (1265 neonates). The inclusion criterion for the study was mothers over 35 years of age with a gestational age of > 28 weeks. The exclusion criteria were: (1) Childbirth at gestational age ≤ 28 weeks by cesarean section or vaginal delivery; (2) Cases of miscarriage, induced labor, or hospitalization for other diseases without delivery.

### Data collection

Data were collected independently by two researchers and reviewed by other researchers after completion. The data collected included the overall condition of the newborn (e.g., gestational age, mode of delivery, presence of fetal distress, NICU transfer), general conditions of the mother (e.g., maternal age, body mass index [BMI], number of pregnancies and births, prenatal screening and diagnostic methods, method of conception, and number of embryos), existing comorbidities before pregnancy (e.g., anemia, scarred uterus, diabetes, chronic hypertension, uterine fibroids, uterine malformations, history of gynecological surgery), pregnancy complications (e.g., placenta previa, placenta abruption, placenta adhesion, placenta implantation, gestational diabetes mellitus [GDM], gestational hypertension [HDCP], preeclampsia [PE], HELLP syndrome, premature rupture of membranes [PPROM], abnormal amniotic fluid, amniotic fluid embolism [AFE], abrupt labor), and other factors (e.g., preterm birth, cause of preterm birth, the conversion from planned vaginal delivery to emergency cesarean section, vaginal birth after a previous cesarean [VBPC]).

### Definitions

In this study, the method of prenatal screening is classified as unknown (type 1), amniocentesis (type 2), no screening (type 3), general Down’s syndrome screening (type 4), and noninvasive DNA screening (type 5). Embryonic number is defined as the number of embryos conceived, with type 1 being a singleton pregnancy and type 2 being a twin pregnancy. PPROM refers to the spontaneous rupture of membranes before 37 weeks of gestation and is classified as absent (No) or present (Yes). PE is a condition that occurs after 20 weeks of gestation. It is characterized by increased blood pressure and proteinuria, as well as symptoms of headache, dizziness, nausea, vomiting, and abdominal discomfort. PE is classified as absent (0), PE (1) and severe PE (2). The HELLP syndrome is a serious pregnancy complication characterized by hemolysis, elevated liver enzymes, and low platelets. Fetal distress is defined as symptoms indicating poor fetal condition before or during birth. Premature birth is the gestational week at which labor occurred: 0 = > 37 weeks, 1 = 34–37 weeks, 2 = < 34 weeks. Causes of preterm birth include none (0), spontaneous (1), and iatrogenic (2). Spontaneous preterm births (SPBs) include spontaneous preterm labor and/or delivery after PPROM. Iatrogenic preterm births (IPBs) include induced labor without PPROM and/or elective cesarean delivery.

NICUs were established successively in China since the 1980s. The criteria for NICU admission are: 1. Circulatory system: (a) life-threatening arrhythmia; (b) invasive hemodynamics monitoring; (c) recovery from heart surgery or unstable heart disease. 2. Respiratory system: (a) assisted breathing; (b) intratracheal respiration; c) > 50% inhaled oxygen concentration. 3. Nervous system: (a) intracranial pressure monitoring; (b) increased intracranial pressure; (c) recovery from intracranial surgery; (d) unstable neurological diseases; (e) closed head trauma. 4. Digestive system: (a) active gastrointestinal bleeding; (b) severe diarrhea and dehydration; (c) recovery from major surgery. 5. Others: (a) application of intravenous medications; (b) severe electrolyte disturbance. In short, any procedure or condition that may worsen at any time and endanger the life of newborns should be treated and monitored in the NICU [6].

### Development and assessment of nomogram

A nomogram was constructed using multivariate logistic regression to predict NICU admission for infants of older mothers. Independent predictors (*P* < 0.1) identified by multivariate logistic regression were included in the construction of the predictive nomogram. Variables were screened by univariate logistic regression, and those with statistical significance (*P* < 0.1) were further analyzed by multivariate stepwise logistic regression. The prediction nomogram was constructed by incorporating independent predictors (*p* < 0.05) identified from multivariate logistic regression and then internally validated using the validation cohort. The predictive accuracy and agreement of the model were assessed using the receiver operating characteristic (ROC) curve, area under the ROC curve (AUC), concordance index (C-index), and calibration curve. The net benefit of the model was evaluated by decision curve analysis (DCA).

### Statistical analysis

All statistical analyses were performed using the *rms*, *mice*, *pROC*, *glmnet*, *recipes*, and *rmda* packages in R version 4.3.1. Missing data (< 5% of cases) were imputed using a multiple-interpolation method. Data are expressed as mean ± standard deviation and compared using the independent sample *t*-test. Count data are represented by n (%) and compared using the chi-squared test. All statistical tests were two-tailed and a *P* < 0.05 was considered statistically significant.

## Results

### General characteristics

**Fig. 1:**
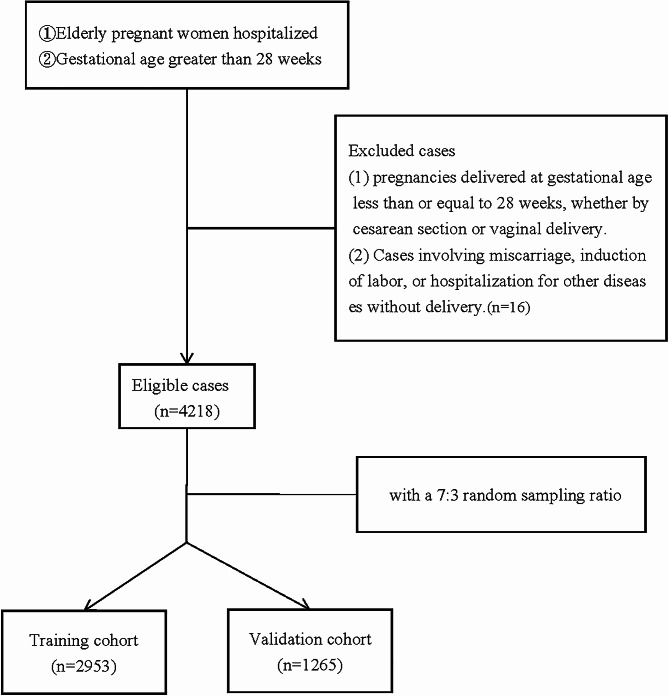
Flow chart for patient selection

The clinical information of 4234 subjects were obtained from the Second Hospital of Shandong University. Sixteen subjects did not meet the inclusion criterion as they were hospitalized for pregnancy complications and discharged without giving birth (Fig. 1). A final total of 4218 subjects were enrolled in this study (2953 in the training cohort and 1265 in the validation cohort). The infants of 337 subjects were transferred to the NICU after birth for further treatment (NICU group) (Table S1). There were no significant differences in BMI, age, gravidity, parity, mode of delivery, primipara, delivery mode, and incidences of scarred uterus, hypertension, uterine fibroids, uterine malformation, placenta previa, placental adhesion, placenta accreta, abnormal amniotic fluid, GDM, AFE, anemia, VBPC, cesarean section after a previous vaginal delivery (CSPVD), and emergency labor between the NICU and control groups (Table S2).

### Screening for predictive factors

Univariate logistic regression revealed 14 perinatal factors (12 with *P* < 0.05, 2 with *P* < 0.1) as potential predictors for NICU admission (Table S2). Subsequent multivariate stepwise regression of these 14 perinatal factors identified 8 perinatal factors to be independent predictors for NICU admission of infants born to older mothers (Table S3). These eight factors were method of prenatal screening, number of implanted embryos, PPROM, PE, HELLP syndrome, fetal distress, premature birth, and cause of preterm birth.

### Construction of prediction nomogram

**Fig. 2:**
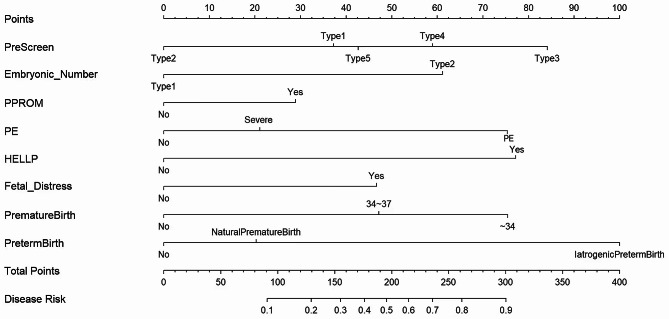
Nomogram for prenatal prediction of NICU admission risk in infants born to mothers of advanced maternal age. Note: The score of each factor is summed to obtain a total score. PreScreen refers to the method of prenatal screening modalities: type 1 = unknown, type 2 = amniocentesis, type 3 = no screening, type 4 = general Down’s syndrome screening, and type 5 = noninvasive DNA screening. Embryonic Number refers to the number of embryos conceived, with type 1 = singleton pregnancy and type 2 = twin pregnancy. PPROM is scored as present (Yes) or absent (No). PE is classified as preeclampsia and severe preeclampsia (Severe). HELLP syndrome is scored as present (Yes) or absent (No). FetalDistress indicates fetal distress, with Yes representing its presence and No representing its absence. PrematureBirth is the gestational week at which labor occurred, including labor between 34 and 37 weeks and labor < 34 weeks. PrematureBirth refers to the cause of preterm labor. NaturePrematureBirth refers to spontaneous preterm births and IatrogenicPretermBirth refers to iatrogenic preterm births

A prediction nomogram was constructed based on the eight perinatal factors using logistic regression (C-index = 0.751, CI: 0.713–0.789) (Fig. 2). In this model, the likelihood of neonatal NICU transfer was calculated based on the total scores of these 8 perinatal factors. As an example, a mother with a singleton pregnancy opts for non-invasive DNA testing to assess the risk of Down’s syndrome. She had severe PE during gestation, fetal distress during delivery, and IPB at 32 weeks of gestation. The corresponding scores for these events were 0, 42.5, 22, 47, 77.5, and 100, respectively. Her total score was 289, which indicates an 87% probability of neonatal NICU transfer.

### Predictive accuracy and net benefit of nomogram

**Fig. 3:**
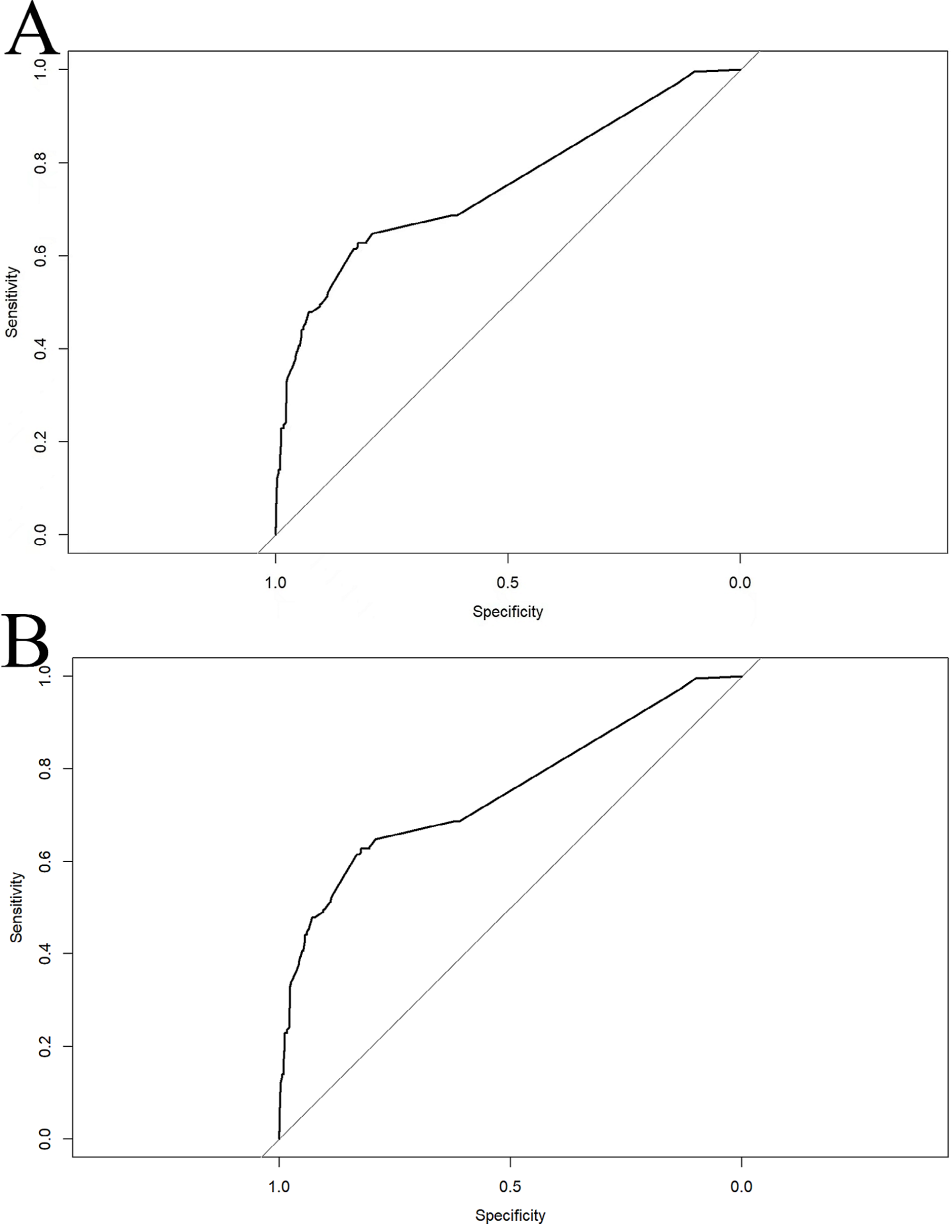
ROC curves of predicted nomogram for the training and validation cohorts. **(A)** Training cohort. **(B)** Validation cohort. Note: The ROC curve is a tool for assessing the performance of a prediction model, where the accuracy, sensitivity, and specificity of the model are calculated by comparing the actual results to the predicted results. The points on the ROC curve indicate the cutoffs for the predicted results, and the closer the area under the ROC curve (AUC) is to 1, the better the predictive performance of the model. In the training cohort, the AUC (95% CI) of this nomogram was 0.7507 (0.714–0.7874) (Fig. **3A**), and the AUC of the validation cohort was 0.7274 (95% CI:0.6787–0.776) (Fig. **3B**)

**Fig. 4:**
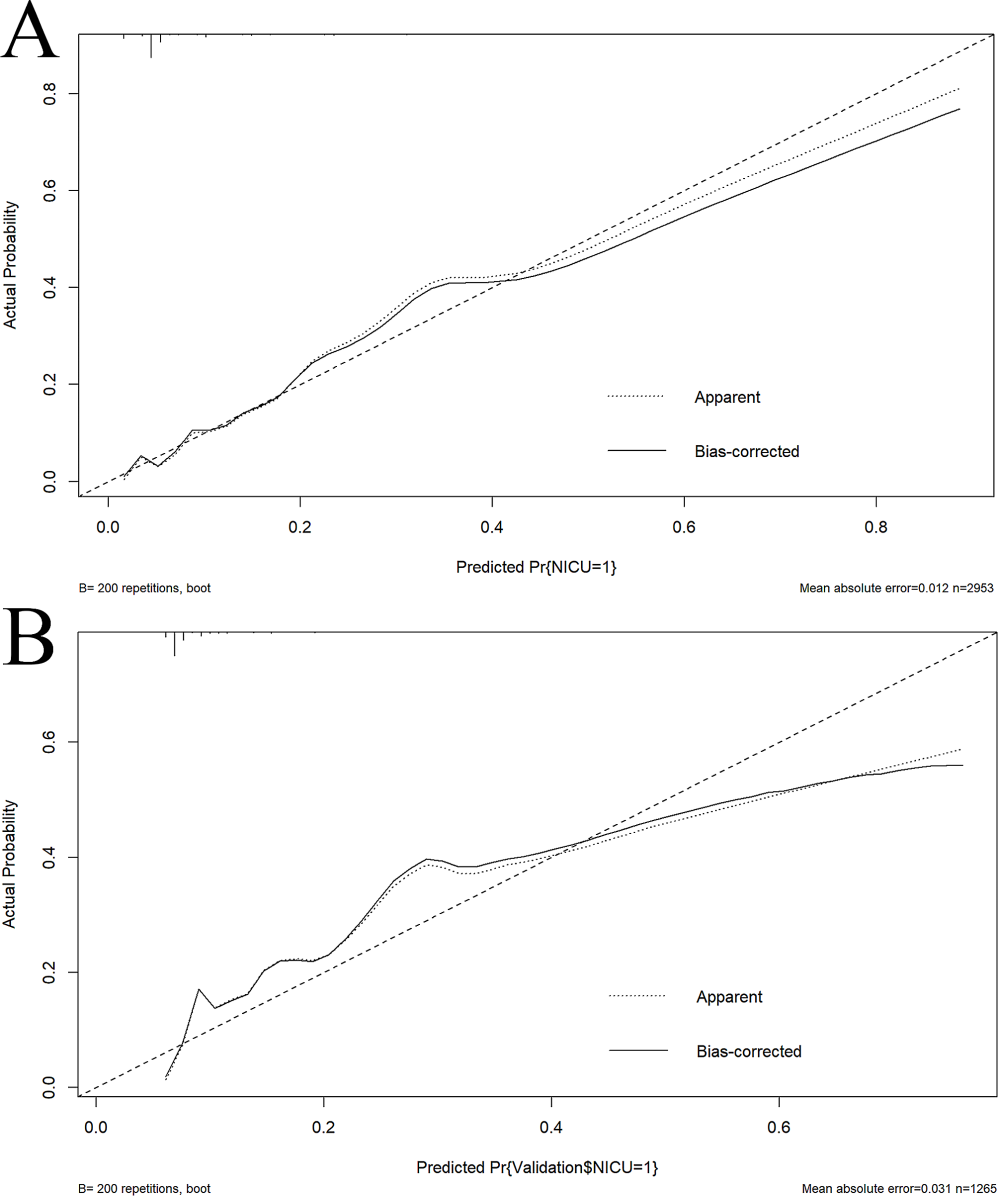
Calibration curves of the training and validation cohorts. **(A)** Training cohort. **(B)** Validation cohort. Note: The calibration curves for both the training cohort (Fig. **4A**) and validation cohort (Fig. **4B**) are very close to the ideal diagonal curve

**Fig. 5:**
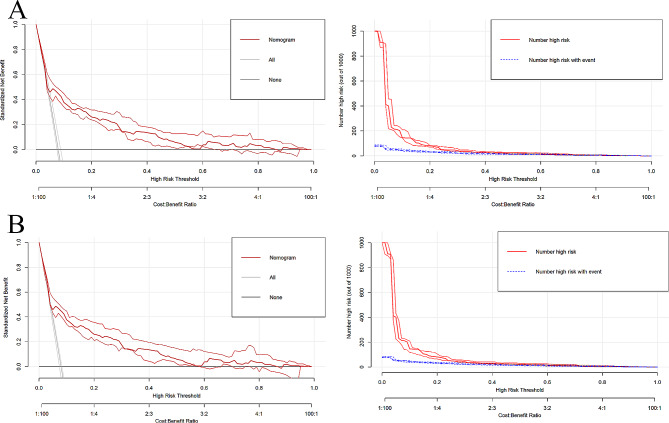
Decision curve and calibration curve for the prediction of neonatal NICU. **(A-1)** Calibration curve of training cohort. **(A-2)** Decision curve of training cohort. **(B-1)** Calibration curve of validation cohort. **(B-2)** Decision curve of validation cohort. Note: Nomogram decision curves and calibration curves for the training and validation cohorts. DCA shows a net benefit decision interval of [0.09,0.59] for the training cohort (Fig. **5A**) and a net benefit decision interval of [0.08,0.58] for the validation cohort (Fig. **5B**). In addition, the clinical decision curves show strong agreement between the predicted high-risk population in the training cohort and the actual high-risk population when risk probability is ≥ 30%. The clinical impact curves for the validation cohort also show that the model accurately identifies high-risk populations when the predicted risk probability is ≥ 30%

In the training cohort, the AUC (95% CI) of the nomogram was 0.7507 (0.714–0.7874) (Fig. 3A). The calibration curve was remarkably close to the ideal diagonal line, and DCA revealed a net benefit decision interval of 0.09 to 0.59 for the training cohort (Fig. 4A). Furthermore, the clinical impact curve showed strong agreement between the predicted high-risk population and the actual high-risk population when the risk probability was ≥ 30% (Fig. 5A). In addition, the model exhibited a high level of consistency, as evidenced by an AUC of 0.7274 (95% CI: 0.6787–0.776) for the validation cohort (Fig. 3B). The calibration curve for the validation cohort was close to the ideal diagonal, and DCA showed a net benefit decision interval of 0.08 to 0.58 (Fig. 4B). Notably, the clinical impact curve of the validation cohort demonstrated that the model accurately identified the high-risk population when the predicted risk probability was ≥ 30% (Fig. 5B).

## Discussion

### Main findings

Pregnancy is a complex process whose outcome can be influenced by both physiological and pathological factors [7]. Identification of factors that can lead to fetal distress is crucial for improving the prognosis of newborns [8]. Preterm birth and maternal complications are the top adverse factors affecting neonatal outcomes [9, 10]. Our study revealed that the method of prenatal screening, number of implanted embryos, PPROM, PE, HELLP syndrome, fetal distress, premature birth, and cause of preterm birth were independent predictors for NICU admission. External validation of the nomogram constructed based on these 8 factors confirmed that the model has a high predictive accuracy, sensitivity, specificity, and clinical utility. Therefore, this nomogram is a simple and useful tool for early prediction of neonatal outcomes.

### Strengths and limitations

Previous studies on neonatal NICU transfer have primarily examined its incidence [11], treatment approaches [12], and subsequent outcomes [13]. However, the novelty of our study stems from the development of a prediction model for neonatal NICU transfer based on perinatal factors. Our results demonstrated that prenatal screening method, number of implanted embryos, PPROM, PE, HELLP syndrome, fetal distress, preterm birth, and cause of preterm birth are independent predictors for neonatal NICU admission following delivery.

There are several limitations in this study. First, newborns born to mothers of AMA have been shown to have a higher risk of adverse outcomes than those born to mothers of normal maternal age [14]. Our study primarily focused on mothers over 35 years of age and did not examine women of normal childbearing age. Second, we did not take into account metabolic and lifestyle factors. Lifestyle varies considerably across regions and countries, and different diet and exercise habits during pregnancy may affect pregnancy outcomes. Therefore, these modeling variables will need to be further investigated in subsequent studies.

### Interpretation (in light of other evidence)

Previous studies have demonstrated that eclampsia [15], HELLP syndrome [16], twin pregnancy [17] with fetal distress [8] during childbirth, PPROM, spontaneous preterm delivery, and iatrogenic preterm delivery [18] are associated with NICU admission. Similarly, we found that these factors, along with the type of prenatal screening, are independent predictors of NICU admission for infants born to mothers of AMA. This emphasizes the importance of educating pregnant individuals on prenatal screening methods to help prevent adverse neonatal outcomes.

In this study, there were five methods of prenatal screening and diagnosis [19], including unknown, amniocentesis, no screening, general Down’s syndrome screening, and non-invasive DNA screening. Our nomogram revealed that the lack of screening posed the highest risk of NICU admission. This suggests that the pregnant mother may not prioritize Down’s syndrome screening or may neglect regular check-ups during pregnancy. Consequently, these individuals are more likely to face complications during pregnancy, and their newborns may be at higher risk of adverse outcomes. In addition, general Down’s syndrome screening posed the second highest risk of NICU admission, possibly due to its limited accuracy [20]. The comparable risk probability between unknown screening and non-invasive DNA screening may be attributed to the fact that pregnant women are sometimes struggle to recall prenatal screening procedures accurately. Even when a screening procedure is done, they may not be able to precisely describe it because of limited familiarity with the procedures. Amniocentesis posed the lowest risk and had the highest predictive accuracy in NICU admission. Consistent with our findings, Martina Provenzano et al. reported that amniocentesis is superior to other methods of prenatal screening in reducing the incidences of trisomy 21 and 18 [21].

It is worth noting that PE has recently been classified as PE without severe features and PE with severe features [22]. Contrary to our initial assumption, our nomogram revealed that newborns of AMA mothers with mild PE had a higher risk of NICU admission. A potential explanation for this finding is that pregnant women with severe PE receive thorough and attentive care during their daily diagnostic and treatment procedures, along with proactive interventions. In contrast, pregnant women with PE without severe features are often due to subtle clinical signs and a lack of noticeable symptoms. Therefore, it is essential to prioritize such pregnant women in clinical practice, especially those at an advanced age.

The number of embryos was also an independent influencing factor for neonatal NICU transfer and was incorporated into our prediction model. It has been well documented that twin pregnancies are associated with higher risk of adverse perinatal and neonatal outcomes compared with singleton pregnancies [23]. PPROM is usually defined as a rupture of fetal membranes before 37 weeks of gestation [24]. It is a major cause of premature birth and perinatal deaths, particularly in developing countries due to poor access and availability of medical resources to manage and sustain the pregnancy to term [25]. HELLP syndrome is a complicated form of severe PE associated with increased risks of maternal and neonatal morbidity and mortality. HELLP syndrome can be definitively cured simply by childbirth [16]. Fetal distress is a widely recognized cause of adverse neonatal outcomes. Persistent fetal distress can easily cause neonatal hypoxemia, hypercapnia, and organ dysfunction [26]. Blood in the body is redistributed through vasoconstriction of blood vessels in non-vital organs (e.g., lungs and intestine) and redirection of blood flow to important organs such as the heart and brain. The lungs are one of the most vulnerable organs susceptible to injuries, such as respiratory distress, pulmonary hemorrhage, persistent pulmonary hypertension, and even respiratory failure [27]. Most adverse outcomes in newborns are associated with preterm birth, which can be further classified as late preterm birth (between 34 and 37 weeks) and early preterm birth (before 34 weeks) [28]. These types of preterm births can be either spontaneous or iatrogenic, with the former accounting for 70-80% of all preterm births [29].

## Conclusion

We developed a prenatal prediction model for NICU admission of neonates born to mothers over 35 years of age. Our validated model demonstrates satisfactory predictive performance and serves as an effective tool for clinicians to quickly assess and predict the risk of NICU admission for newborns born to mothers of AMA. This is crucial for enhancing doctor-patient communication and alleviating the psychological burden on mothers and families.

### Electronic supplementary material

Below is the link to the electronic supplementary material.

Supplementary Material 1

Supplementary Material 2

## Data Availability

All data generated or analyzed during this study are included in this published article.
