# Supplementary material for: Development and validation of a prenatal predictive nomogram for the risk of NICU admission in infants born to Chinese mothers over 35 years of age: a retrospective cohort study
Source: BMC Pregnancy Childbirth. 2024 May 27;24:390. doi: 10.1186/s12884-024-06582-0 (PMC11129413; doi:10.1186/s12884-024-06582-0)

**Supplementary material 1:** Research ethics committee approval of the Second Hospital of Shangdong University.（KYLL-2023LW051）


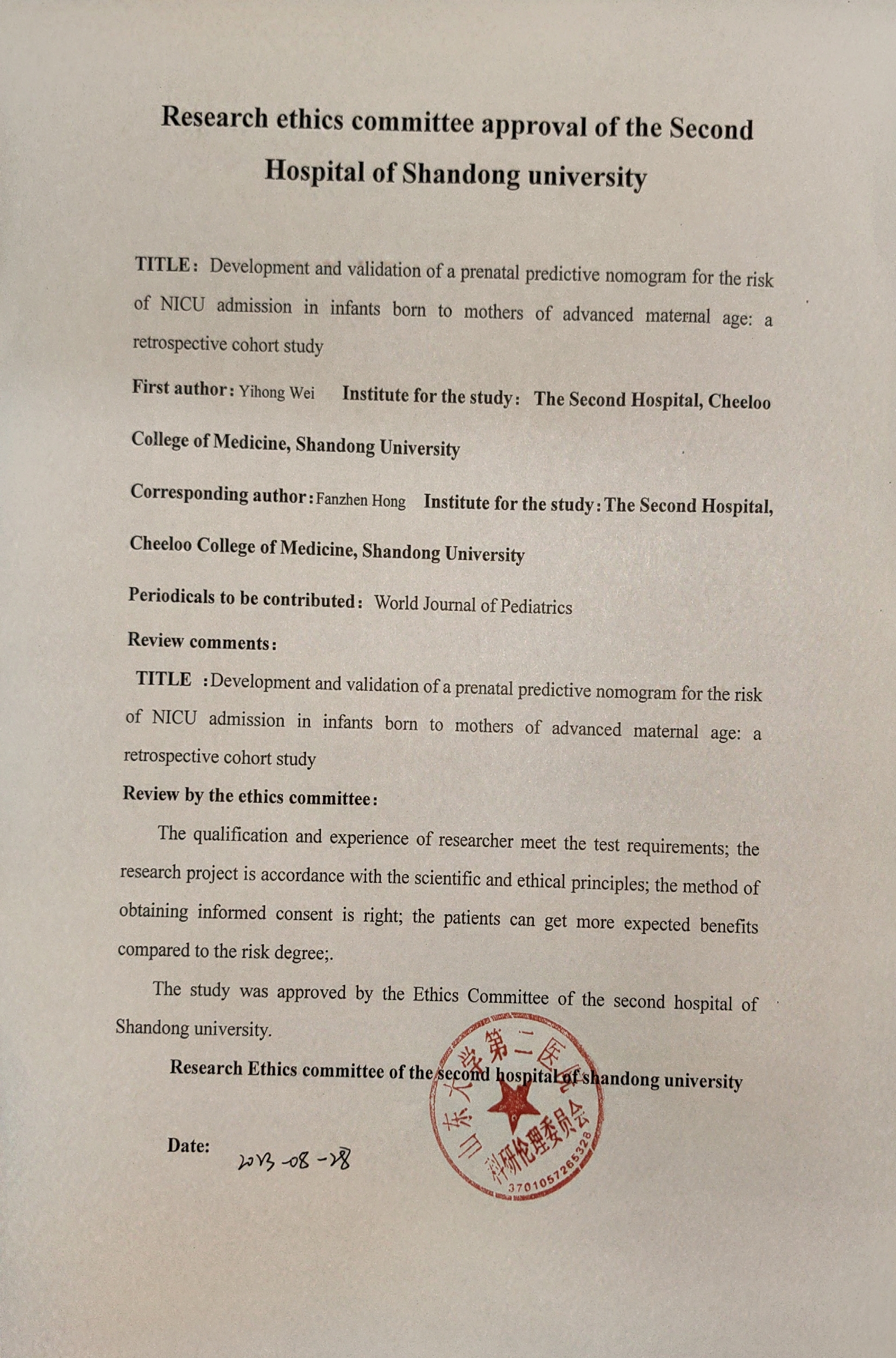

Supplement: Supplementary file 1 — Supplementary Material 1 [file 12884_2024_6582_MOESM1_ESM.docx]
