# Supplementary material for: Development and validation of a prenatal predictive nomogram for the risk of NICU admission in infants born to Chinese mothers over 35 years of age: a retrospective cohort study
Source: BMC Pregnancy Childbirth. 2024 May 27;24:390. doi: 10.1186/s12884-024-06582-0 (PMC11129413; doi:10.1186/s12884-024-06582-0)
Supplement: Supplementary file 2 — Supplementary Material 2 [file 12884_2024_6582_MOESM2_ESM.docx]

**Table S1** Baseline characteristics of all patients in the training cohort and validation cohort.

| **No** | **Factors** | **value** | **Training(n=2953)** | **Validation(n=1265)** | **Overall(n=4218)** | **t/x2** | **P** |
| --- | --- | --- | --- | --- | --- | --- | --- |
| 1 | Group |  |  |  |  |  |  |
| 2 | BMI |  | 29.14±5.51 | 29.44±8.22 | 29.23±6.44 | -1.366 | 0.172 |
| 3 | Age |  | 37.65±2.3 | 37.68±2.35 | 37.66±2.32 | -0.348 | 0.728 |
| 4 | Gravidity |  |  |  |  | 6.185 | 0.626 |
|  |  | 1 | 92(3.1) | 50(4) | 142(3.4) |  |  |
|  |  | 2 | 727(24.6) | 331(26.2) | 1058(25.1) |  |  |
|  |  | 3 | 937(31.7) | 389(30.8) | 1326(31.4) |  |  |
|  |  | 4 | 734(24.9) | 312(24.7) | 1046(24.8) |  |  |
|  |  | 5 | 318(10.8) | 124(9.8) | 442(10.5) |  |  |
|  |  | 6 | 112(3.8) | 41(3.2) | 153(3.6) |  |  |
|  |  | 7 | 21(0.7) | 9(0.7) | 30(0.7) |  |  |
|  |  | 8 | 9(0.3) | 7(0.6) | 16(0.4) |  |  |
|  |  | 9 | 3(0.1) | 2(0.2) | 5(0.1) |  |  |
| 5 | Parity（Times） |  |  |  |  | 4.603 | 0.023 |
|  |  | 1 | 208(7) | 104(8.2) | 312(7.4) |  |  |
|  |  | 2 | 2338(79.2) | 973(76.9) | 3311(78.5) |  |  |
|  |  | 3 | 380(12.9) | 170(13.4) | 550(13) |  |  |
|  |  | 4 | 27(0.9) | 18(1.4) | 45(1.1) |  |  |
| 6 | PreScreen |  |  |  |  | 2.697 | 0.61 |
|  |  | 1 | 142(4.8) | 60(4.7) | 202(4.8) |  |  |
|  |  | 2 | 345(11.7) | 134(10.6) | 479(11.4) |  |  |
|  |  | 3 | 10(0.3) | 5(0.4) | 15(0.4) |  |  |
|  |  | 4 | 622(21.1) | 249(19.7) | 871(20.6) |  |  |
|  |  | 5 | 1834(62.1) | 817(64.6) | 2651(62.8) |  |  |
| 7 | Pregnancy_Mode |  |  |  |  | 0.148 | 0.701 |
|  |  | 0 | 2877(97.4) | 1235(97.6) | 4112(97.5) |  |  |
|  |  | 1 | 76(2.6) | 30(2.4) | 106(2.5) |  |  |
| 8 | Embryonic_Number |  |  |  |  | 0.202 | 0.653 |
|  |  | 0 | 2928(99.2) | 1256(99.3) | 4184(99.2) |  |  |
|  |  | 1 | 25(0.8) | 9(0.7) | 34(0.8) |  |  |
| 9 | Primiparous |  |  |  |  | 1.793 | 0.181 |
|  |  | 0 | 2745(93) | 1161(91.8) | 3906(92.6) |  |  |
|  |  | 1 | 208(7) | 104(8.2) | 312(7.4) |  |  |
| 10 | Scarred_Uterus |  |  |  |  | 2.662 | 0.103 |
|  |  | 0 | 1619(54.8) | 728(57.5) | 2347(55.6) |  |  |
|  |  | 1 | 1334(45.2) | 537(42.5) | 1871(44.4) |  |  |
| 11 | Hypertension |  |  |  |  | 0.022 | 0.811 |
|  |  | 0 | 2857(96.7) | 1225(96.8) | 4082(96.8) |  |  |
|  |  | 1 | 96(3.3) | 40(3.2) | 136(3.2) |  |  |
| 12 | PreDiabetes |  |  |  |  | 0.004 | 0.948 |
|  |  | 0 | 2922(99) | 1252(99) | 4174(99) |  |  |
|  |  | 1 | 31(1) | 13(1) | 44(1) |  |  |
| 13 | Uterine_Fibroids |  |  |  |  | 0.853 | 0.356 |
|  |  | 0 | 2719(92.1) | 1154(91.2) | 3873(91.8) |  |  |
|  |  | 1 | 234(7.9) | 111(8.8) | 345(8.2) |  |  |
| 14 | Uterine_Malformation |  |  |  |  | 1.204 | 0.273 |
|  |  | 0 | 2936(99.4) | 1261(99.7) | 4197(99.5) |  |  |
|  |  | 1 | 17(0.6) | 4(0.3) | 21(0.5) |  |  |
| 15 | HGSurgery |  |  |  |  | 0.122 | 0.727 |
|  |  | 0 | 2158(73.1) | 931(73.6) | 3089(73.2) |  |  |
|  |  | 1 | 795(26.9) | 334(26.4) | 1129(26.8) |  |  |
| 16 | Placenta_Previa |  |  |  |  | 0.002 | 0.963 |
|  |  | 0 | 2894(98) | 1240(98) | 4134(98) |  |  |
|  |  | 1 | 59(2) | 25(2) | 84(2) |  |  |
| 17 | Placental_Abruption |  |  |  |  | 0.09 | 0.764 |
|  |  | 0 | 2922(99) | 1253(99.1) | 4175(99) |  |  |
|  |  | 1 | 31(1) | 12(0.9) | 43(1) |  |  |
| 18 | Placental_Adhensions |  |  |  |  | 0.45 | 0.502 |
|  |  | 0 | 2845(96.3) | 1224(96.8) | 4069(96.5) |  |  |
|  |  | 1 | 108(3.7) | 41(3.2) | 149(3.5) |  |  |
| 19 | Placenta_Accreta |  |  |  |  | 2.222 | 0.136 |
|  |  | 0 | 2937(99.5) | 1253(99.1) | 4190(99.3) |  |  |
|  |  | 1 | 16(0.5) | 12(0.9) | 28(0.7) |  |  |
| 20 | PPROM |  |  |  |  | 0.203 | 0.653 |
|  |  | 0 | 2796(94.7) | 1202(95) | 3998(94.8) |  |  |
|  |  | 1 | 157(5.3) | 63(5) | 220(5.2) |  |  |
| 21 | PROM |  |  |  |  | 0.013 | 0.909 |
|  |  | 0 | 2400(81.3) | 1030(81.4) | 3430(81.3) |  |  |
|  |  | 1 | 553(18.7) | 235(18.6) | 788(18.7) |  |  |
| 22 | Abnormal Amniotic |  |  |  |  | 3.11 | 0.211 |
|  |  | 0 | 2680(90.8) | 1126(89) | 3806(90.2) |  |  |
|  |  | 1 | 209(7.1) | 105(8.3) | 314(7.4) |  |  |
|  |  | 2 | 64(2.2) | 34(2.7) | 98(2.3) |  |  |
| 23 | GDM |  |  |  |  | 1.511 | 0.219 |
|  |  | 0 | 2912(98.6) | 1241(98.1) | 4153(98.5) |  |  |
|  |  | 1 | 41(1.4) | 24(1.9) | 65(1.5) |  |  |
| 24 | HDCP |  |  |  |  | 0.018 | 0.892 |
|  |  | 0 | 2507(84.9) | 1076(85.1) | 3583(84.9) |  |  |
|  |  | 1 | 446(15.1) | 189(14.9) | 635(15.1) |  |  |
| 25 | PE |  |  |  |  | 1.381 | 0.501 |
|  |  | 0 | 2794(94.6) | 1200(94.9) | 3994(94.7) |  |  |
|  |  | 1 | 54(1.8) | 17(1.3) | 71(1.7) |  |  |
|  |  | 2 | 105(3.6) | 48(3.8) | 153(3.6) |  |  |
| 26 | HELLP |  |  |  |  | 0.096 | 0.756 |
|  |  | 0 | 2941(99.6) | 1259(99.5) | 4200(99.6) |  |  |
|  |  | 1 | 12(0.4) | 6(0.5) | 18(0.4) |  |  |
| 27 | AFE |  |  |  |  | 0.016 | 0.899 |
|  |  | 0 | 2951(99.9) | 1264(99.9) | 4215(99.9) |  |  |
|  |  | 1 | 2(0.1) | 1(0.1) | 3(0.1) |  |  |
| 28 | Anemia |  |  |  |  | 0.567 | 0.451 |
|  |  | 0 | 1857(62.9) | 780(61.7) | 2637(62.5) |  |  |
|  |  | 1 | 1096(37.1) | 485(38.3) | 1581(37.5) |  |  |
| 29 | Fetal_Distress |  |  |  |  | 5.208 | 0.022 |
|  |  | 0 | 2663(90.2) | 1111(87.8) | 3774(89.5) |  |  |
|  |  | 1 | 290(9.8) | 154(12.2) | 444(10.5) |  |  |
| 30 | DOGB |  | 267.88±15.1 | 268.23±15.32 | 267.99±15.17 | -0.693 | 0.489 |
| 31 | Delivery mode |  |  |  |  | 0.01 | 0.918 |
|  |  | 0 | 971(32.9) | 418(33) | 1389(32.9) |  |  |
|  |  | 1 | 1982(67.1) | 847(67) | 2829(67.1) |  |  |
| 32 | VBAC |  |  |  |  | 0.339 | 0.56 |
|  |  | 0 | 2940(99.6) | 1261(99.7) | 4201(99.6) |  |  |
|  |  | 1 | 13(0.4) | 4(0.3) | 17(0.4) |  |  |
| 33 | VBCTCS |  |  |  |  | 0.058 | 0.81 |
|  |  | 0 | 2916(98.7) | 1248(98.7) | 4164(98.7) |  |  |
|  |  | 1 | 37(1.3) | 17(1.3) | 54(1.3) |  |  |
| 34 | Emergency_Labor |  |  |  |  | 0.23 | 0.632 |
|  |  | 0 | 2739(92.8) | 1168(92.3) | 3907(92.6) |  |  |
|  |  | 1 | 214(7.2) | 97(7.7) | 311(7.4) |  |  |
| 35 | PrematureBirth |  |  |  |  | 0.448 | 0.8 |
|  |  | 0 | 2611(88.4) | 1117(88.3) | 3728(88.4) |  |  |
|  |  | 1 | 230(7.8) | 95(7.5) | 325(7.7) |  |  |
|  |  | 2 | 112（3.8） | 53（4.2） | 165（3.9） |  |  |
| 36 | PretermBirth |  |  |  |  | 0.953 | 0.621 |
|  |  | 0 | 2657(90) | 1142(90.3) | 3799(90.1) |  |  |
|  |  | 1 | 236(8) | 93(7.4) | 329(7.8) |  |  |
|  |  | 2 | 60（2） | 30（2.4） | 90（2.1） |  |  |
| 37 | NICU |  |  |  |  | 0 | 0.993 |
|  |  | 0 | 2717(92) | 1164(92) | 3881(92) |  |  |
|  |  | 1 | 236(8) | 101(8) | 337(8) |  |  |

**Notes:** Gravidity is the number of pregnancies; Parity (Times) the number of deliveries; PreScreen is the type of prenatal screening, the first (Type1) is unknown, the second (Type2) is amniocentesis, the third (Type3) is no screening, the fourth (Type4) is general Down's screening, and the fifth (Type5) is noninvasive DNA screening;Pregnancy Mode: 0=natural delivery, 1=cesarean section; Embryonic Number:0=single pregnancy, 1=twin pregnancy; Abnormal Amniotic: 0=normal, 1=too little, 2=too much; PE: 0=none, 1=PE, 2=severe; PrematureBirth: 0=> 37 weeks, 1=34-37 weeks, 2=< 34 weeks; PretermBirth: 0=no preterm birth, 1=natural premature birth, 2=iatrogenic preterm birth. Among the other factors, 0= no and 1= yes. HGSurgery=History of gynecological surgery; PPROM=preterm premature rupture of membrane; PROM=premature rupture of membrane; GDM=gestational diabetes mellitus; HDCP: hypertensive disorder complicating pregnancy; PE=preeclampsia; HELLP=hemolysis, elevated liver enzymes, and a low platelet; AFE=amniotic fluid embolism; DOGB=Days of giving birth; VBAC=Vaginal Birth After Cesarean; VBCTCS=Vaginal birth cesarean transferred to cesarean section.

.

**Table S2** Differences between NICU and non-NICU in the training cohort.

| **No** | **Factors** | **value** | **None-NICU(n=)** | **NICU(n=)** | **Overall(n=2953)** | **t/x2** | **P** |
| --- | --- | --- | --- | --- | --- | --- | --- |
| 1 | Group |  |  |  |  |  |  |
| 2 | BMI |  | 29.12±5.64 | 29.43±3.66 | 29.14±5.51 | -0.841 | 0.4 |
| 3 | Age |  | 37.66±2.3 | 37.59±2.38 | 37.65±2.30 | 0.42 | 0.675 |
| 4 | Gravidity |  |  |  |  | 4.24 | 0.835 |
|  |  | 1 | 84(3.1) | 8(3.4) | 92(3.1) |  |  |
|  |  | 2 | 676(24.9) | 51(21.6) | 727(24.6) |  |  |
|  |  | 3 | 858(31.6) | 79(33.5) | 937(31.7) |  |  |
|  |  | 4 | 671(24.7) | 63(26.7) | 734(24.9) |  |  |
|  |  | 5 | 297(10.9) | 21(8.9) | 318(10.8) |  |  |
|  |  | 6 | 100(3.7) | 12(5.1) | 112(3.8) |  |  |
|  |  | 7 | 20(0.7) | 1(0.4) | 21(0.7) |  |  |
|  |  | 8 | 8(0.3) | 1(0.4) | 9(0.3) |  |  |
|  |  | 9 | 3(0.1) | 0(0) | 3(0.1) |  |  |
| 5 | Parity（Times） |  |  |  |  | 2.535 | 0.469 |
|  |  | 1 | 186(6.8) | 22(9.3) | 208(7) |  |  |
|  |  | 2 | 2155(79.3) | 183(77.5) | 2338(79.2) |  |  |
|  |  | 3 | 352(13) | 28(11.9) | 380(12.9) |  |  |
|  |  | 4 | 24(0.9) | 3(1.3) | 27(0.9) |  |  |
| 6 | PreScreen |  |  |  |  | 19.783 | 0.001 |
|  |  | 1 | 132(4.9) | 10(4.2) | 142(4.8) |  |  |
|  |  | 2 | 332(12.2) | 13(5.5) | 345(11.7) |  |  |
|  |  | 3 | 9(0.3) | 1(0.4) | 10(0.3) |  |  |
|  |  | 4 | 550(20.2) | 72(30.5) | 622(21.1) |  |  |
|  |  | 5 | 1694(62.3) | 140(59.3) | 1834(62.1) |  |  |
| 7 | Pregnancy_Mode |  |  |  |  | 0.681 | 0.409 |
|  |  | 0 | 2649(97.5) | 228(96.6) | 2877(97.4) |  |  |
|  |  | 1 | 68(2.5) | 8(3.4) | 76(2.6) |  |  |
| 8 | Embryonic_Number |  |  |  |  | 54.885 | 0 |
|  |  | 0 | 2704(99.5) | 224(94.9) | 2928(99.2) |  |  |
|  |  | 1 | 13(0.5) | 12(5.1) | 25(0.8) |  |  |
| 9 | Primiparous |  |  |  |  | 2.034 | 0.154 |
|  |  | 0 | 2531(93.2) | 214(90.7) | 2745(93) |  |  |
|  |  | 1 | 186(6.8) | 22(9.3) | 208(7) |  |  |
| 10 | Scarred_Uterus |  |  |  |  | 0.036 | 0.85 |
|  |  | 0 | 1491(54.9) | 128(54.2) | 1619(54.8) |  |  |
|  |  | 1 | 1226(45.1) | 108(45.8) | 1334(45.2) |  |  |
| 11 | Hypertension |  |  |  |  | 0.016 | 0.9 |
|  |  | 0 | 2629(96.8) | 228(96.6) | 2857(96.7) |  |  |
|  |  | 1 | 88(3.2) | 8(3.4) | 96(3.3) |  |  |
| 12 | PreDiabetes |  |  |  |  | 2.821 | 0.093 |
|  |  | 0 | 2691(99) | 231(97.9) | 2922(99) |  |  |
|  |  | 1 | 26(1) | 5(2.1) | 31(1) |  |  |
| 13 | Uterine_Fibroids |  |  |  |  | 0.107 | 0.744 |
|  |  | 0 | 2503(92.1) | 216(91.5) | 2719(92.1) |  |  |
|  |  | 1 | 214(7.9) | 20(8.5) | 234(7.9) |  |  |
| 14 | Uterine_Malformation |  |  |  |  | 0.331 | 0.565 |
|  |  | 0 | 2702(99.4) | 234(99.2) | 2936(99.4) |  |  |
|  |  | 1 | 15(0.6) | 2(0.8) | 17(0.6) |  |  |
| 15 | HGSurgery |  |  |  |  | 3.115 | 0.078 |
|  |  | 0 | 1974(72.7) | 184(78) | 2158(73.1) |  |  |
|  |  | 1 | 743(27.3) | 52(22) | 795(26.9) |  |  |
| 16 | Placenta_Previa |  |  |  |  |  |  |
|  |  | 0 | 2663(98) | 231(97.9) | 2894(98) | 0.019 | 0.89 |
|  |  | 1 | 54(2) | 5(2.1) | 59(2) |  |  |
| 17 | Placental_Abruption |  |  |  |  | 18.862 | 0 |
|  |  | 0 | 2695(99.2) | 227(96.2) | 2922(99) |  |  |
|  |  | 1 | 22(0.8) | 9(3.8) | 31(1) |  |  |
| 18 | Placental_Adhensions |  |  |  |  | 0.348 | 0.555 |
|  |  | 0 | 2616(96.3) | 229(97) | 2845(96.3) |  |  |
|  |  | 1 | 101(3.7) | 7(3) | 108(3.7) |  |  |
| 19 | Placenta_Accreta |  |  |  |  | 0.445 | 0.505 |
|  |  | 0 | 2703(99.5) | 234(99.2) | 2937(99.5) |  |  |
|  |  | 1 | 14(0.5) | 2(0.8) | 16(0.5) |  |  |
| 20 | PPROM |  |  |  |  | 74.063 | 0 |
|  |  | 0 | 2601(95.7) | 195(82.6) | 2796(94.7) |  |  |
|  |  | 1 | 116(4.3) | 41(17.4) | 157(5.3) |  |  |
| 21 | PROM |  |  |  |  | 7.559 | 0.006 |
|  |  | 0 | 2224(81.9) | 176(74.6) | 2400(81.3) |  |  |
|  |  | 1 | 493(18.1) | 60(25.4) | 553(18.7) |  |  |
| 22 | Abnormal Amniotic |  |  |  |  | 3.302 | 0.192 |
|  |  | 0 | 2470(90.9) | 210(89) | 2680(90.8) |  |  |
|  |  | 1 | 192(7.1) | 17(7.2) | 209(7.1) |  |  |
|  |  | 2 | 55(2) | 9(3.8) | 64(2.2) |  |  |
| 23 | GDM |  |  |  |  | 0.176 | 0.675 |
|  |  | 0 | 2680(98.6) | 232(98.3) | 2912(98.6) |  |  |
|  |  | 1 | 37(1.4) | 4(1.7) | 41(1.4) |  |  |
| 24 | HDCP |  |  |  |  | 33.098 | 0 |
|  |  | 0 | 2337(86) | 170(72) | 2507(84.9) |  |  |
|  |  | 1 | 380(14) | 66(28) | 446(15.1) |  |  |
| 25 | PE |  |  |  |  | 58.144 | 0 |
|  |  | 0 | 2596(95.5) | 198(83.9) | 2794(94.6) |  |  |
|  |  | 1 | 42(1.5) | 12(5.1) | 54(1.8) |  |  |
|  |  | 2 | 79(2.9) | 26(11) | 105(3.6) |  |  |
| 26 | HELLP |  |  |  |  | 4.74 | 0.029 |
|  |  | 0 | 2708(99.7) | 233(98.7) | 2941(99.6) |  |  |
|  |  | 1 | 9(0.3) | 3(1.3) | 12(0.4) |  |  |
| 27 | AFE |  |  |  |  | 0.174 | 0.677 |
|  |  | 0 | 2715(99.9) | 236(100) | 2951(99.9) |  |  |
|  |  | 1 | 2(0.1) | 0(0) | 2(0.1) |  |  |
| 28 | Anemia |  |  |  |  | 1.456 | 0.228 |
|  |  | 0 | 1700(62.6) | 157(66.5) | 1857(62.9) |  |  |
|  |  | 1 | 1017(37.4) | 79(33.5) | 1096(37.1) |  |  |
| 29 | Fetal_Distress |  |  |  |  | 24.767 | 0 |
|  |  | 0 | 2472(91) | 191(80.9) | 2663(90.2) |  |  |
|  |  | 1 | 245(9) | 45(19.1) | 290(9.8) |  |  |
| 30 | DOGB |  | 268.91±13.91 | 256.04±21.84 | 267.88±15.10 | 8.897 | 0 |
| 31 | Delivery mode |  |  |  |  | 1.206 | 0.272 |
|  |  | 0 | 901(33.2) | 70(29.7) | 971(32.9) |  |  |
|  |  | 1 | 1816(66.8) | 166(70.3) | 1982(67.1) |  |  |
| 32 | VBAC |  |  |  |  | 0.002 | 0.968 |
|  |  | 0 | 2705(99.6) | 235(99.6) | 2940(99.6) |  |  |
|  |  | 1 | 12(0.4) | 1(0.4) | 13(0.4) |  |  |
| 33 | VBCTCS |  |  |  |  | 0.341 | 0.559 |
|  |  | 0 | 2682(98.7) | 234(99.2) | 2916(98.7) |  |  |
|  |  | 1 | 35(1.3) | 2(0.8) | 37(1.3) |  |  |
| 34 | Emergency_Labor |  |  |  |  | 0.575 | 0.448 |
|  |  | 0 | 2523(92.9) | 216(91.5) | 2739(92.8) |  |  |
|  |  | 1 | 194(7.1) | 20(8.5) | 214(7.2) |  |  |
| 35 | PrematureBirth |  |  |  |  | 315.766 | 0 |
|  |  | 0 | 2482(91.4) | 129(54.7) | 2611(88.4) |  |  |
|  |  | 1 | 171(6.3) | 59(25) | 230(7.8) |  |  |
|  |  | 2 | 64(2.4） | 48(20.3） | 112(3.8) |  |  |
|  |  |  |  |  |  |  |  |
| 36 | PretermBirth |  |  |  |  | 402.817 | 0 |
|  |  | 0 | 2520(92.7) | 137(58.1) | 2657(90) |  |  |
|  |  | 1 | 177(6.5) | 59(25) | 236(8) |  |  |
|  |  | 2 | 20(0.7) | 40(16.9) | 60(2) |  |  |

**Note:** Gravidity is the number of pregnancies; Parity (Times) the number of deliveries; PreScreen is the type of prenatal screening, the first (Type1) is unknown, the second (Type2) is amniocentesis, the third (Type3) is no screening, the fourth (Type4) is general Down's screening, and the fifth (Type5) is noninvasive DNA screening; Pregnancy_Mode is the mode of conception: 0=natural conception, 1=assisted reproduction; Embryonic_Number is the number of embryos: 0=single pregnancy, 1=twin pregnancy. PPROM=preterm premature rupture of membrane; PROM=premature rupture of membrane; GDM=gestational diabetes mellitus; HDCP=hypertensive disorder complicating pregnancy; PE=preeclampsia; HELLP=HELLP syndrome, which means hemolysis, elevated liver enzymes, and a low platelet; DOGB=Days of giving birth; VBAC=Vaginal Birth After Cesarean; Abnormal Amniotic: 0=Normal, 1=Low Amniotic Fluid, 2=Excessive Amniotic Fluid; PE (Pre-eclampsia): 0=None, 1=Slightly, 2=Severe; PrematureBirth means the weeks of labor: 0=weeks of labor > 37 weeks, 1=weeks of labor between 34 and 37 weeks, 2=weeks of labor < 34 weeks; PreternBirth is the cause of preterm labor: 0=no preterm labor, 1=spontaneous preterm birth, 2=iatrogenic preterm birth. For the remaining factors, 0=No, 1=Yes.

**Table S3** Results of the analysis of independent risk factors for admission of infants to the NICU.

| **Factors** | **B** | **se** | **chi-square** | **P** | **OR** | **OR(95% CI)** | |
| --- | --- | --- | --- | --- | --- | --- | --- |
|  |  |  |  |  |  | **low** | **up** |
| PreScreen |  |  | 14.855 | 0.005 |  |  |  |
| PreScreen(1) | -0.115 | 0.373 | 0.095 | 0.758 | 0.892 | 0.430 | 1.851 |
| PreScreen(2) | -0.895 | 0.317 | 7.980 | 0.005 | 0.409 | 0.220 | 0.760 |
| PreScreen(3) | 0.870 | 1.067 | 0.664 | 0.415 | 2.386 | 0.295 | 19.323 |
| PreScreen(4) | 0.342 | 0.175 | 3.809 | 0.051 | 1.408 | 0.999 | 1.985 |
| Embryonic_Number(1) | -1.282 | 0.483 | 7.057 | 0.008 | 0.277 | 0.108 | 0.715 |
| PPROM(1) | -0.607 | 0.257 | 5.575 | 0.018 | 0.545 | 0.329 | 0.902 |
| PE |  |  | 19.517 | 0.000 |  |  |  |
| PE(1) | -0.442 | 0.326 | 1.840 | 0.175 | 0.643 | 0.339 | 1.217 |
| PE(2) | 1.138 | 0.486 | 5.474 | 0.019 | 3.120 | 1.203 | 8.092 |
| HELLP(1) | -1.619 | 0.774 | 4.371 | 0.037 | 0.198 | 0.043 | 0.904 |
| Fetal_Distress(1) | -0.979 | 0.203 | 23.325 | 0.000 | 0.376 | 0.252 | 0.559 |
| PrematureBirth |  |  | 11.331 | 0.003 |  |  |  |
| PrematureBirth(1) | -1.582 | 0.476 | 11.053 | 0.001 | 0.206 | 0.081 | 0.522 |
| PrematureBirth(2) | -0.592 | 0.278 | 4.543 | 0.033 | 0.553 | 0.321 | 0.954 |
| PretermBirth |  |  | 26.542 | 0.000 |  |  |  |
| PretermBirth(1) | -2.098 | 0.497 | 17.846 | 0.000 | 0.123 | 0.046 | 0.325 |
| PretermBirth(2) | -1.672 | 0.353 | 22.391 | 0.000 | 0.188 | 0.094 | 0.375 |
| Constant | 5.405 | 1.029 | 27.589 | 0.000 | 222.515 |  |  |
